# A Facile Approach for the Preparation of Nano-size Zinc Oxide in Water/Glycerol with Extremely Concentrated Zinc Sources

**DOI:** 10.1186/s11671-018-2616-0

**Published:** 2018-07-09

**Authors:** Zhiguo Wang, Hongwei Li, Fanghua Tang, Jinxia Ma, Xiaofan Zhou

**Affiliations:** Jiangsu Co-Innovation Center of Efficient Processing and Utilization of Forest Resources, Nanjing Forestry University, Nanjing, 210037 China

**Keywords:** Nano-ZnO, Glycerol, Zinc chloride

## Abstract

A facile process to prepare zinc oxide (ZnO) nanoparticles from an aqueous zinc chloride (ZnCl_2_) solution and an aqueous hydroxide solution under a glycerol stabilizer at room temperature was developed. ZnCl_2_ aqueous solutions as concentrated as 65–80 wt% were used as the concentrated zinc source. The concentration of ZnCl_2_ solutions and the molar ratio of glycerol to Zn^2+^ had obvious effects on the sizes and shapes of the ZnO nanoparticles. The shape of ZnO nanoparticles changed from rods approximately 50–120 nm long and 30–70 nm in diameter to globular with diameters of approximately 20 nm with the increasing of the concentration of the ZnCl_2_ solution and the mole ratio of glycerol to Zn^2+^. Glycerol, as a stabilizer, played an important role in the formation of ZnO nanostructures at room temperature, even for a highly concentrated zinc source.

## Background

Zinc oxide (ZnO) nanoparticles are one of the most important multifunctional semiconductor materials for applications in electronic and optoelectronic devices [1], solar cells [2], field-emission devices [3], sensors [4], and photocatalysts [5]. ZnO nanoparticles are also generally recognized as safe and biocompatible and have been used as drug carriers and medical filling materials [6], photoluminescence agents in biosensors [7], UV-absorbers in sun creams and coating materials [8], and antibacterial agents in many industrial products [9, 10].

Many methods have been developed to synthesize ZnO nanoparticles with different sizes and morphologies, including chemical vapor deposition [11], sol-gel methods [12], hydrothermal methods [13], laser ablation [14], microemulsion techniques [15], and others [16]. Due to their large specific surface areas as well as high surface energies, ZnO nanoparticles tend to agglomerate easily. Most approaches require the use of stabilizers and low concentrations of ZnO precursors [17–20]. Thus, most processes require a large amount of water or organic solvents. Furthermore, hard agglomerates regularly appear with the use of water in synthesis processes, which presents an obstacle to the application of ZnO nanoparticles [21]. The polyol approach was shown to be suitable for the preparation of metal oxide nanoparticles [22]. ZnO nanoparticles have been successfully synthesized in various polyol media such as ethylene glycol (EG) [23], diethylene glycol (DEG) [24], 1,3-propanediol (PD) [25], tetraethylene glycol (TEG) [23], and 1,4-butanediol (BD) [26]. The nucleation and growth of ZnO nanoparticles were carried out in a high boiling polyol. The polyol serves as both solvent and stabilizing agent to prevent the agglomeration of nanoparticles. Chieng and Loo fabricated ZnO nanoparticles of different sizes and shapes by refluxing 1 mol/L (M) zinc acetate (Zn(CH_3_COO)_2_) in EG, DEG, and TEG at 160 °C for 12 h. They found the average particle size of synthesized ZnO increased with increasing glycol chain length. The shape of the ZnO nanoparticles changed from spherical (EG), to spherical and rod (DEG), to ‘diamond’-like structures (TEG) [23]. Mezni et al. prepared ZnO nanoparticles with a mean diameter of 5 nm using 1,3-propanediol as a solvent and 1.2 mM Zn(CH_3_COO)_2_ as a precursor at 160 °C for 1 h. 1,3-propanediol also plays the roles of stabilizer and template [25].The drawbacks of the polyol process are the low concentrations of ZnO precursors and high temperatures of reaction systems.

ZnCl_2_ is a highly soluble salt in water; its solubility is 432 g/100 g water at 25 °C (highest concentration up to 81.2 wt%) [27]. Moreover, it is a ZnO precursor. If nano-sized ZnO could be obtained from a concentrated ZnCl_2_ aqueous solution, many problems might be solved, such as the formation of hard agglomerates and the use of an aqueous environment. In our previous study, ZnO nanoparticles could be prepared via in situ synthesis of ZnO in dissolved starch or cellulose by a highly concentrated ZnCl_2_ aqueous solution (such as 65 wt% of ZnCl_2_ aqueous solution) [28, 29]. However, in such processes, starch or cellulose would need to be dissolved by concentrated ZnCl_2_ aqueous solution at ~ 80 °C for approximately 2 h. To make the synthesis process more easily performed in one step, with a high concentration of ZnO precursors at room temperature and in a short reaction time, glycerol was used as a stabilizer in a highly concentrated ZnCl_2_ aqueous solution to generate ZnO nanoparticles in this study. Glycerol is an environmentally friendly polyol that has high solubility in water. However, it has been rarely employed in the synthesis of ZnO nanoparticles [22–26, 30]. Therefore, our study focuses on the correlations between ZnO nanoparticle size and morphology as well as the concentration of ZnCl_2_ solutions, mole ratios of glycerol/Zn^2+^, and types of hydroxides. Furthermore, at present, little attention has been paid to a simple route for fabricating ZnO nanoparticles in a water/glycerol system, where water acts as a solvent and glycerol acts as both stabilizer and template. The synthesis was easily performed in one step with high concentrations of ZnO precursors (ZnCl_2_ and NaOH aqueous solutions up to 80 and 50 wt% in water, respectively) and under mild reaction conditions such as room temperature and a short reaction time (10 min). Moreover, the size and morphology of ZnO nanoparticles could be controlled by the amount of glycerol and the concentration of ZnCl_2_ solutions.

## Methods/Experimental

### Materials

ZnCl_2_, NaOH, KOH, LiOH, ammonia water, and glycerol of analytical grade (Nanjing chemical reagent factory, China) were used without further purification.

### Preparation of the ZnO Nanoparticle

First, glycerol was added to ZnCl_2_ aqueous solutions with a certain mole ratios of glycerol to Zn^2+^. Then an alkaline solution was added dropwise to the ZnCl_2_-glycerol solution at room temperature under continuous mechanical agitation to achieve a final pH value of 12, following which the reaction continued for 5 min, the conditions of preparation of the ZnO nanoparticles was seen Table 1. At the end of the reaction, a white emulsion was generated. The white emulsions were washed twice with water and ethanol, respectively, and centrifuged (6000 rpm, 10 min). After drying in an oven at 80 °C, the ZnO nanoparticles were obtained.

**Table 1:** The conditions of preparation of the ZnO nanoparticles

Mole ratio of glycerol to Zn^2+^	Concentration of ZnCl_2_ solution (wt%)	Hydroxides	Concentration of hydroxide (wt%)
0:1	65	NaOH	50
0.33:1	65	NaOH	50
1:1	65	NaOH	50
1.67:1	65	NaOH	50
3.33:1	65	NaOH	50
1:1	50	NaOH	50
1:1	65	NaOH	50
1:1	80	NaOH	50
3.33:1	65	LiOH	8
3.33:1	65	NH_4_OH	25
3.33:1	65	NaOH	50
3.33:1	65	KOH	60

### Characterization of ZnO Nanoparticles

The X-ray diffraction patterns (XRD) were recorded using an X-ray diffractometer (Ultima IV, Japan). The morphology of the ZnO nanoparticles was investigated by a scanning electron microscope (SEM) (JSM-7600F; JEOL, Tokyo, Japan) and a transmission electron microscope (TEM) (JEM-2100, JEOL, Japan). An X-ray photoelectron spectroscopy (XPS) (AXIS Ultra DLD system, UK) was used to identify the chemical bonding states of the Zn and O. The UV spectrum of the ZnO nanoparticles was recorded with a UV-visible spectrophotometer (Lambda 950, Perkin Elmer, USA), and the maximum excitation wavelength was 325 nm.

## Results and Discussion

### The Influence of the Mole Ratio of Glycerol to Zn^2+^ on ZnO Nanoparticles Size and Morphology

**Fig. 1:**
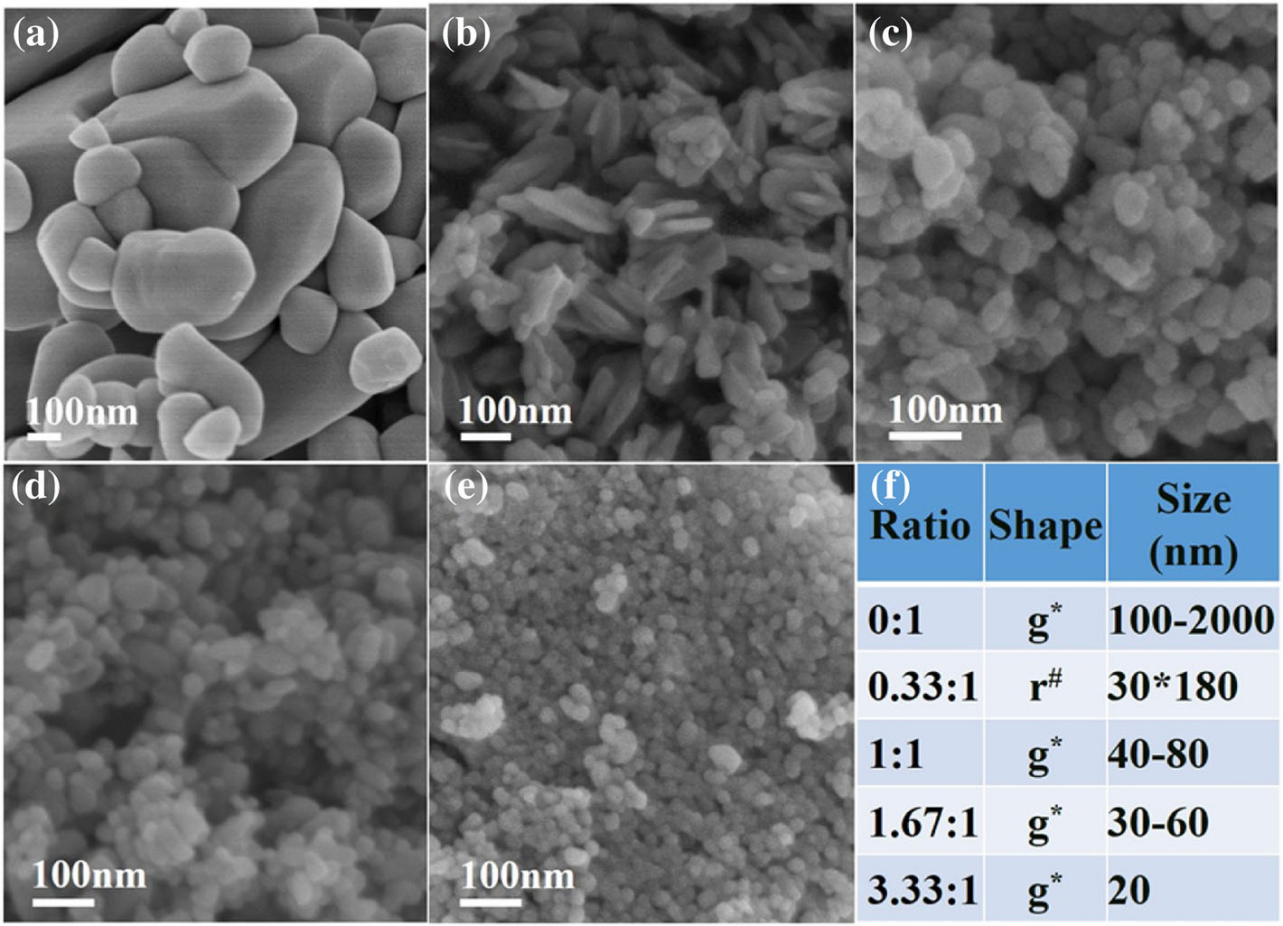
SEM images of ZnO nanoparticles obtained from 65% ZnCl_2_ aqueous solution under the different mole ratios of glycerol to Zn^2+^ (**a**, 0:1; **b**, 0.33:1; **c**, 1:1; **d**, 1.67:1; **e**, 3.33:1) and (**f**) the statistics table about the morphology and size of ZnO nanoparticles (g^*^: globular, r^#^: rod)

First, the role of glycerol in the synthesis of ZnO nanoparticles was studied. The influence of the mole ratio of glycerol to Zn^2+^ on the morphology of ZnO nanoparticles was investigated. Figure 1 demonstrates the effect of the mole ratio of glycerol to Zn^2+^ on the morphology of ZnO nanoparticles, as the reactants were 65 wt% ZnCl_2_ and 50 wt% NaOH in water. Apparently, the sizes of ZnO nanoparticles prepared without glycerol (Fig. 1a) were much larger than those prepared with glycerol stabilizer (Fig. 1b–e) under the same concentration of ZnCl_2_. The ZnO nanoparticles prepared without glycerol were shown to be heterogeneous (Fig. 1a). These results indicate that glycerol, as a stabilizer, played an important role in the formation of ZnO nanostructures. When the mole ratio of glycerol to Zn^2+^ was 0.33, the obtained ZnO consisted of a few globular particles and many ZnO rods, approximately 180 nm in length and 30 nm in diameter; the aspect ratio of the rod-shaped ZnO nanoparticles was approximately 6 (Fig. 1b). When the mole ratio of glycerol to Zn^2+^ increased to 1 and 1.67, the obtained ZnO was globular, with approximately 40–80 nm and 30–60 nm diameters, respectively (Fig. 1c and Fig. 1d). In addition, uniform and globular ZnO nanoparticles with a diameter of approximately 20 nm were obtained at the 3.33 mol ratio of glycerol to Zn^2+^ (Fig. 1e). It may be inferred that glycerol played an important role in the synthesis procedure described in this work.

**Fig. 2:**
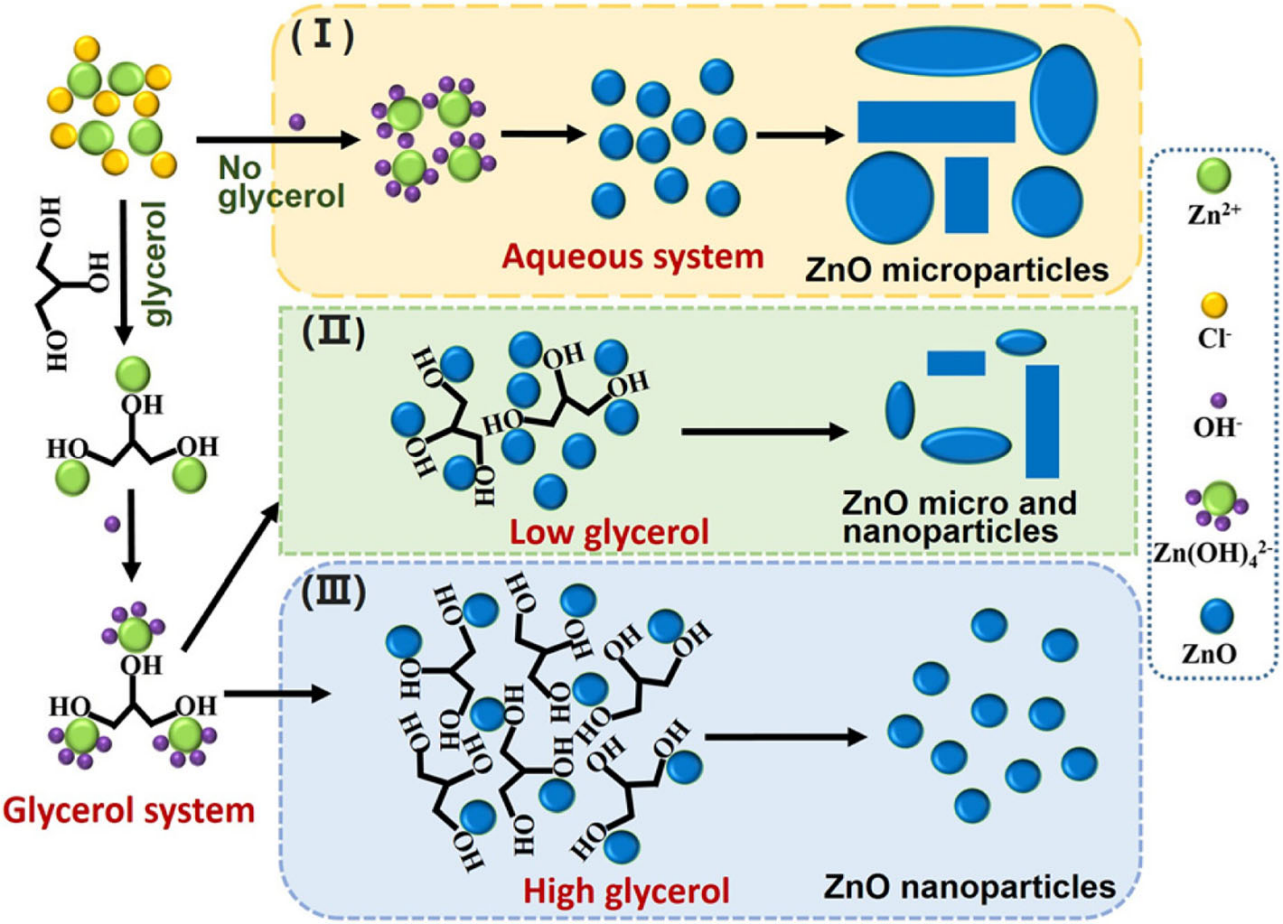
Schematic presentation of glycerol role in the synthesis process of ZnO nanoparticles

When the concentration of zinc ions and OH^−^ increase without stabilizer, supersaturated ZnO nuclei would more easily aggregate, growing larger and heterogeneous ZnO nanoparticles (seen from Fig. 2(I)). Glycerol has three hydroxyl groups, with which the zinc ions could interact to form a zinc-glycerol complex. When NaOH solution is added, the zinc-glycerol complex reacts with OH^−^ to form Zn(OH)_4_^2−^ around the hydroxyl groups of glycerol. Then, Zn(OH)_4_^2−^ dehydrates into ZnO near the glycerol. Meanwhile, the high NaOH concentration (50 wt%) causes a burst of initial homogeneous nucleation of ZnO crystals, and the supersaturated ZnO nuclei aggregate together near the glycerol, which acts as a stabilizer. As seen from Fig. 2(II), at a low glycerol content in the glycerol/water system, the blocking effect of glycerol was reduced, meaning that since less glycerol is preventing the growth and agglomeration of ZnO in the reaction system, the resulting ZnO nanoparticles were uneven and had a larger size. Meanwhile, with a high glycerol content, the combination of the hydroxyl groups of glycerol and zinc ions greatly reduced the concentration of unbonded zinc ions. The increase of the blocking effect of glycerol causes ZnO to be much more uniform and to exhibit the smallest size (seen from Fig. 2(III)). In fact, the morphologies of ZnO could be controlled by the mole ratio of glycerol to Zn^2+^. In brief, glycerol operates as an obstructer of agglomeration and a stabilizer of nano-sized ZnO under condition of an extremely concentrated zinc source and an alkaline solution.

**Fig. 3:**
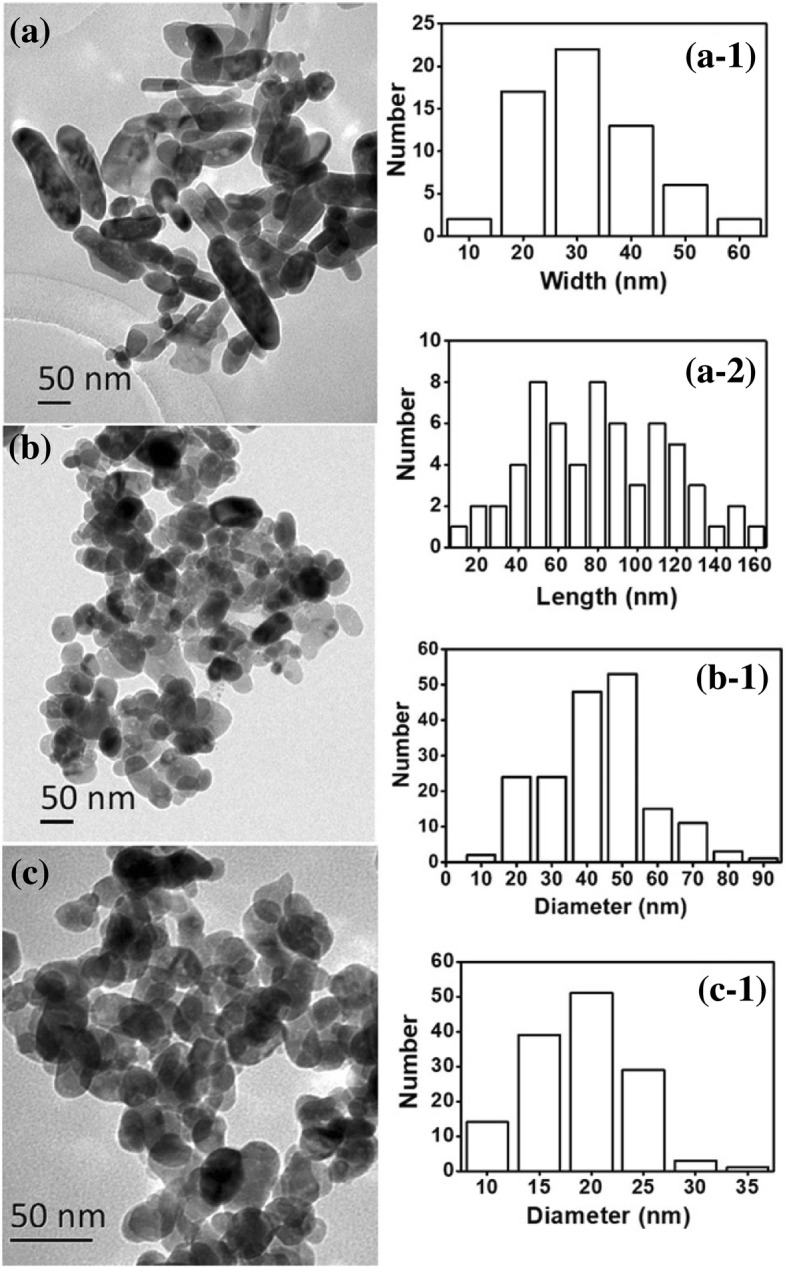
TEM images of ZnO nanoparticles obtained from the different mole ratios of glycerol to Zn^2+^ (**a**, 0.33:1; **b**, 1:1; **c**, 3.33:1). a-1, a-2 corresponding histogram of (**a**) sample; b-1, c-1 corresponding histogram of (**b**) and (**c**) sample respectively

ZnO nanoparticles were characterized by TEM, XPS, XRD, and UV-visible spectrophotometry. Figure 3 illustrates that mostly ZnO rods (with some globular particles) were obtained from the 0.33 mol ratio of glycerol to Zn^2+^ at a concentration of 65 wt% ZnCl_2_. The ZnO rods had a wide range of lengths from 20 to 160 nm, namely, due to the uneven directional growth that appeared in the process. Globular ZnO particles with a diameter of approximately 40–50 nm were obtained when the mole ratio of glycerol to Zn^2+^ was 1 and the concentration of ZnCl_2_ was 65 wt% in aqueous solution. Furthermore, uniform and globular ZnO nanoparticles with a diameter of approximately 15–25 nm were obtained from the 3.33 of mole ratio of glycerol to Zn^2+^ when the concentration of ZnCl_2_ aqueous solution was 65 wt%. These results were consistent with the SEM results (Fig. 1). It was further confirmed that glycerol has an important effect on the preparation of ZnO nanoparticles. Moreover, ZnO nanoparticles could be generated in the presence of glycerol using a relatively highly concentrated ZnCl_2_ aqueous solution at room temperature.

**Fig. 4:**
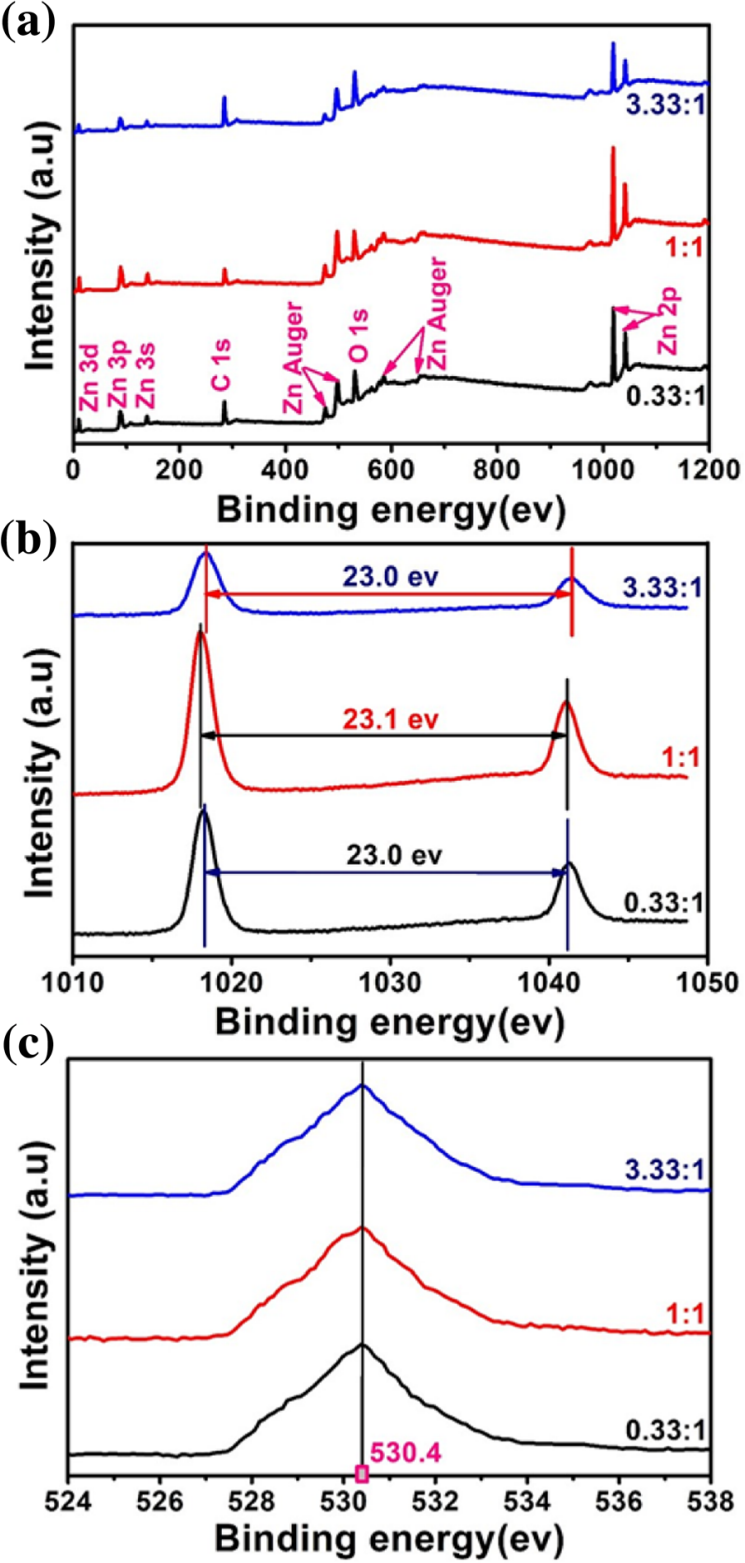
XPS spectra of ZnO nanoparticles obtained from the different mole ratios of glycerol to Zn^2+^ (**a**, wide; **b**, Zn-2p; **c**, O-1s)

The element composition and chemical bond of the ZnO samples were further analyzed by XPS technique, as shown in Fig. 4. Zn 3d, Zn 3p, Zn 3 s, Zn Auger, and Zn 2p peaks besides the C1s and O1s peaks were identified in Fig. 4a. The existence of C1s peak demonstrated little residual glycerol in the three ZnO nanoparticles. The Zn 2p spectrum shows a doublet (Fig. 4b), which can be identified as the Zn 2p3/2 and Zn 2p1/2 lines, respectively. The binding energy differences between the two lines are 23.0 eV (from mole ratio of glycerol to Zn^2+^ 0.33:1 and 3.33:1) or 23.1 eV (from mole ratio of glycerol to Zn^2+^ 1:1), which confirms that the Zn atoms are in a completely oxidized state in all samples. Figure 4c shows the O1s high-resolution XPS spectra of ZnO. For rod or globular ZnO particles, the peaks all exhibited at around 530.4 eV is attributed to the oxidized metal ions in the nanoparticles, namely, O-Zn in the ZnO lattice.

**Fig. 5:**
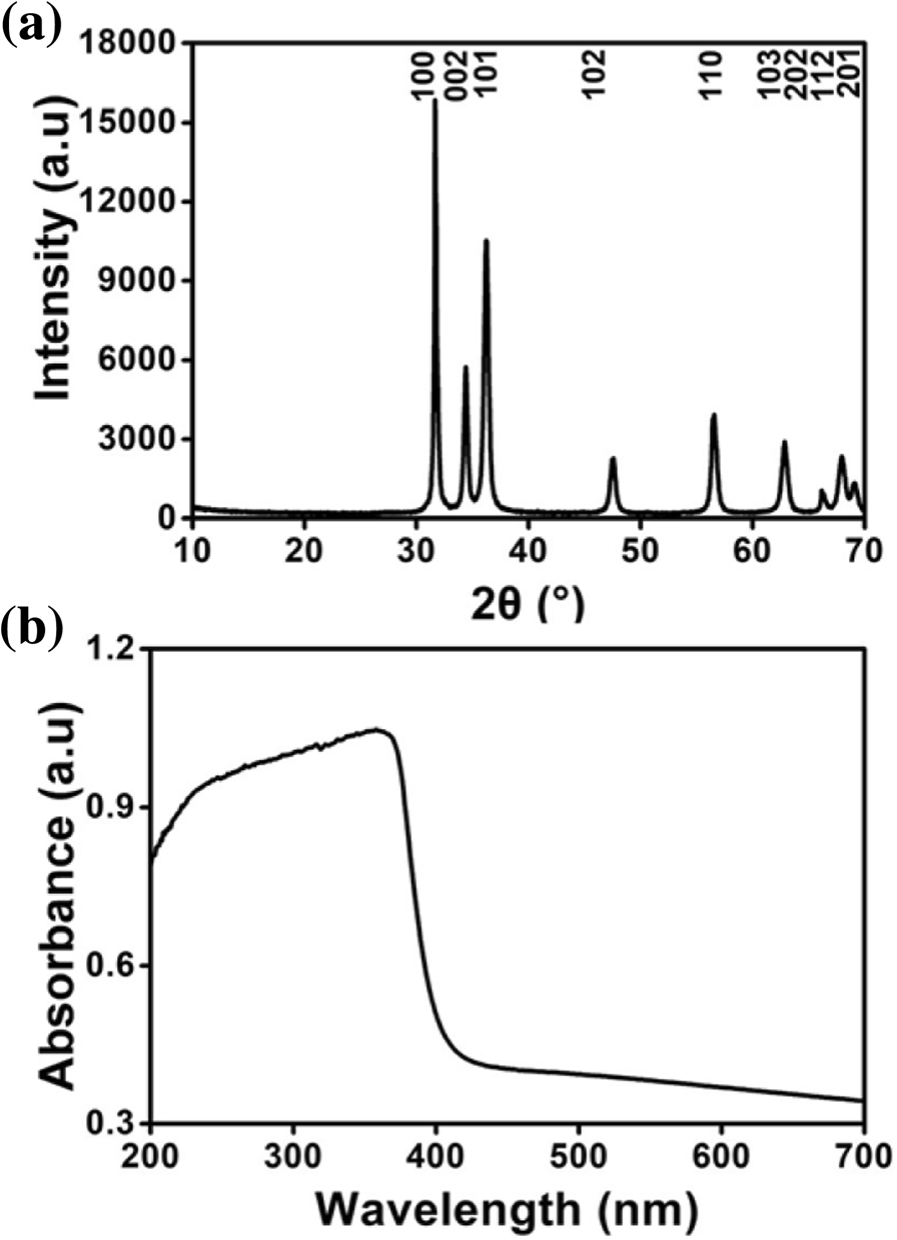
XRD pattern (**a**) and UV-vis absorption spectrum (**b**) of ZnO nanoparticles

Only diffraction peaks typical of the wurtzite ZnO crystal structure can be seen in Fig. 5a. The UV-vis absorption spectra of the ZnO nanoparticles are shown in Fig. 5b. The ZnO nanoparticles exhibited broad and strong absorption, with a maximum at approximately 380 nm. The figure shows that pure nano-sized ZnO could be prepared from a concentrated zinc source and a glycerol stabilizer and template. Furthermore, the ZnO nanoparticles possess UV blocking properties. Therefore, the ZnO nanoparticles prepared from our approach have a potential application in sun creams or coating materials.

### The Influence of the Concentration of ZnCl_2_ Aqueous Solutions on ZnO Nanoparticle Size and Morphology

Globular ZnO nanoparticles could be prepared via 65 and 50 wt% ZnCl_2_ and NaOH aqueous solutions, respectively, at a 1:1 mol ratio of glycerol to Zn^2+^ (Fig. 1c). To investigate the influence of concentration of ZnCl_2_ on ZnO nanoparticle size and morphology, 50 wt% of NaOH solution and a 1:1 mol ratio of glycerol to Zn^2+^ were employed.

**Fig. 6:**
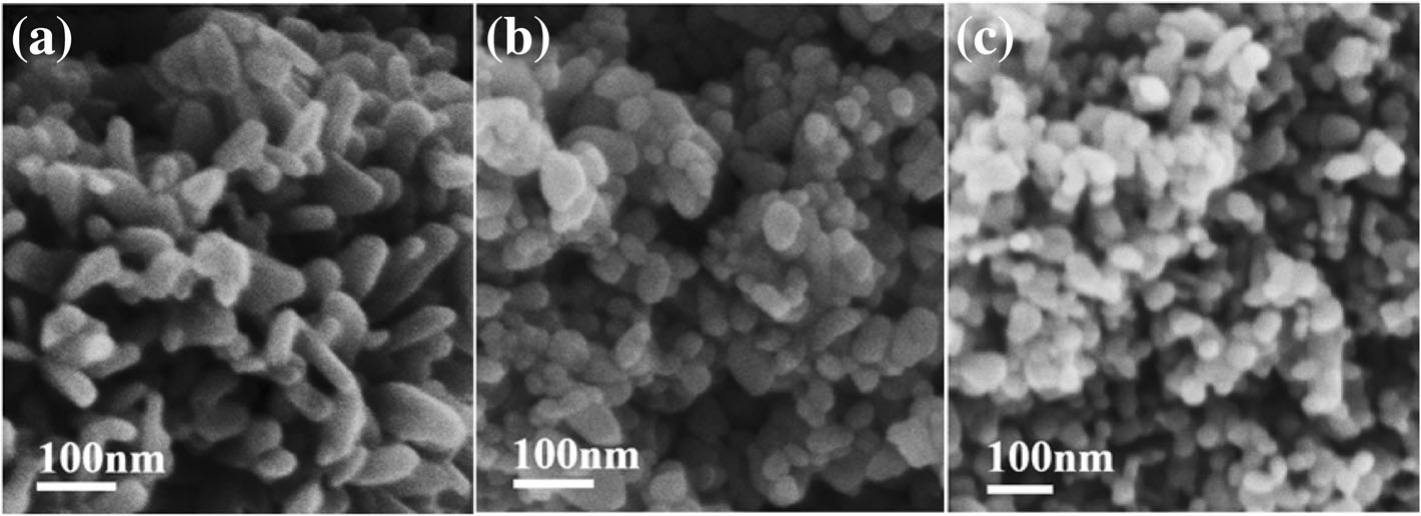
SEM images of ZnO nanoparticles obtained from the different concentration of ZnCl_2_ aqueous solution under the 1:1 of mole ratio of glycerol to Zn^2+^ (**a**, 50 wt%; **b**, 65 wt%; **c**, 80 wt%)

Figure 6 shows that the concentration of ZnCl_2_ in solution had an obvious effect on the shapes and sizes of ZnO nanoparticles. The shape of ZnO changed from rods to globular as the concentration of ZnCl_2_ increased under obstruction with glycerol as a stabilizer. In the presented approach, the shape of ZnO nanoparticles was changed, and the particle size decreased when the concentration of ZnCl_2_ aqueous solution was increased (in other words, the hydration ratio was decreased). The results obtained in this system present glycerol as a stabilizer because, otherwise, the homogeneous ZnO nanoparticle cannot be prepared from such a highly concentrated zinc source (see Fig. 1a). For a 50 wt% concentration of ZnCl_2_ (hydration ratio of 7.56), the obtained ZnO rods were approximately 130 nm in length and 30–70 nm in diameter (Fig. 6a). When the concentration of the ZnCl_2_ aqueous solution increased to 65 wt% (hydration ratio decreased to 4.07), the obtained ZnO was globular, with an approximately 40–80 nm diameter (Fig. 6b). In addition, as shown in Fig. 6c, uniform and globular ZnO nanoparticles with a diameter of approximately 40 nm were obtained from 80 wt% ZnCl_2_ aqueous solution (hydration ratio of 1.89). In fact, the morphologies of ZnO could be controlled by the concentration of ZnCl_2_ aqueous solution (or the hydration ratio). The results are consistent with those of Poul et al. [31]. However, in their polyol process, DEG served as a solvent, and a low concentration of ZnO precursors (lower than 0.3 M) at the DEG boiling point was used. Moreover, uneven and larger ZnO nanoparticles were fabricated in the absence of glycerol at a 65 wt% concentration of ZnCl_2_ (Fig. 1a). In this study, the ZnO nanoparticle was obtained using relatively highly concentrated (80 wt%, namely, 29.3 M) ZnCl_2_ at room temperature. In addition, in the presence of glycerol, the size of ZnO nanoparticles decreased as the concentration of ZnCl_2_ aqueous solution increased, which disagreed with the previous results. It may be that as the concentration of ZnCl_2_ was increasing (less water) in our approach, the interaction abilities of zinc ions and the hydroxyl oxygens of glycerol increased, meaning that the blocking effect of glycerol was enhanced, resulting in smaller ZnO nanoparticles.

### The Influence of the Hydroxides on ZnO Nanoparticles Size and Morphology

The impact of the types of hydroxides on ZnO nanoparticles size and morphology were also investigated under obstruction with glycerol as stabilizer. The objective was to prepare ZnO nanoparticles with the highest concentration of substances in this study. Therefore, saturated solution of NaOH, KOH, LiOH, and NH_4_OH were prepared at room temperature: LiOH at 8 wt% (3.63 M), NaOH at 50 wt% (25 M), KOH at 60 wt% (26.74 M), and ammonia at 25 wt% (9.51 M). Meanwhile, to generate more even and smaller ZnO nanoparticles, a 3.33:1 mol ratio of glycerol to Zn^2+^ was employed.

**Fig. 7:**
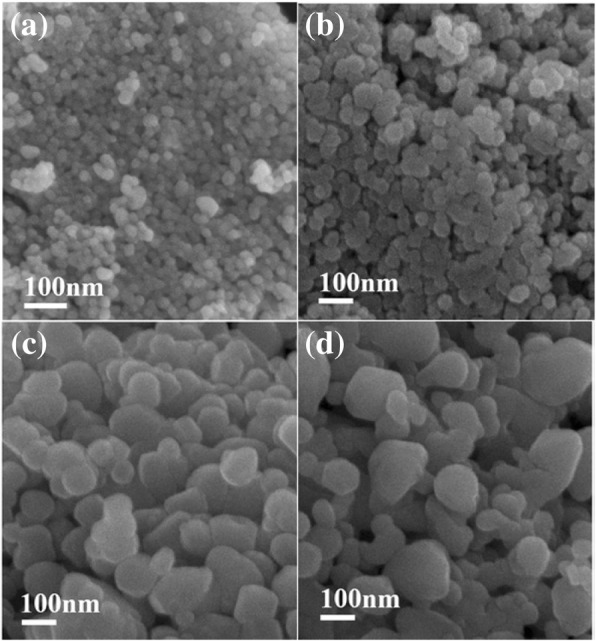
SEM images of ZnO nanoparticles obtained from 65% ZnCl_2_ aqueous solution by reactivity with various hydroxides (**a**, NaOH; **b**, KOH; **c**, LiOH; **d**, NH_4_OH)

Figure 7 shows SEM images of ZnO nanoparticles obtained from 65% ZnCl_2_ aqueous solution by reaction with various hydroxides. The results indicated that the hydroxides had an obvious influence on the size of ZnO nanoparticles. The ZnO nanoparticles obtained from NaOH, KOH, LiOH, and NH_4_OH were all granular, and the sizes of ZnO nanoparticles were approximately 20 nm from NaOH, 50 nm from KOH, 80–150 nm from LiOH, and 50–300 nm from NH_4_OH, respectively. It could be proposed that due to high concentrations of ZnCl_2_ solution (65 wt%) and hydroxides, the formation of ZnO was very fast during the early nucleation stage, generating many ZnO nuclei near glycerol. Meanwhile, the cations, such as Na^+^, K^+^, Li^+^, or NH_4_^+^, could provide a passivating layer around the ZnO surface, slowing the growth of ZnO nanoparticles and preventing particle agglomeration. The radii sequence of hydrated cationic is Li^+^ > Na^+^ > NH_4_^+^ > K^+^. Higher concentrations of K^+^ or NH_4_^+^ than Li^+^ or Na^+^ were required to provide the near-complete passivation on the ZnO surfaces. Additionally, the saturation concentrations of LiOH and ammonia water were 3.63 and 9.51 M, which are much lower than those of NaOH (25 M), and KOH (26.74 M). The amount of Li^+^ and NH_4_^+^ could not provide the near-complete passivation on the ZnO surfaces, inhibiting any further growth of ZnO nanoparticles. In addition, the concentrations of LiOH and ammonia water were low, meaning that more water reduced the interaction abilities of zinc ions and the hydroxyl oxygens of the glycerol, resulting simultaneously in the reduction of the blocking effect of glycerol. Therefore, ZnO nanoparticles obtained from LiOH and NH_4_OH were bigger.

### Preparation of ZnO Nanoparticles Obstruction by Glycerol, Starch, or Cellulose Systems

**Fig. 8:**
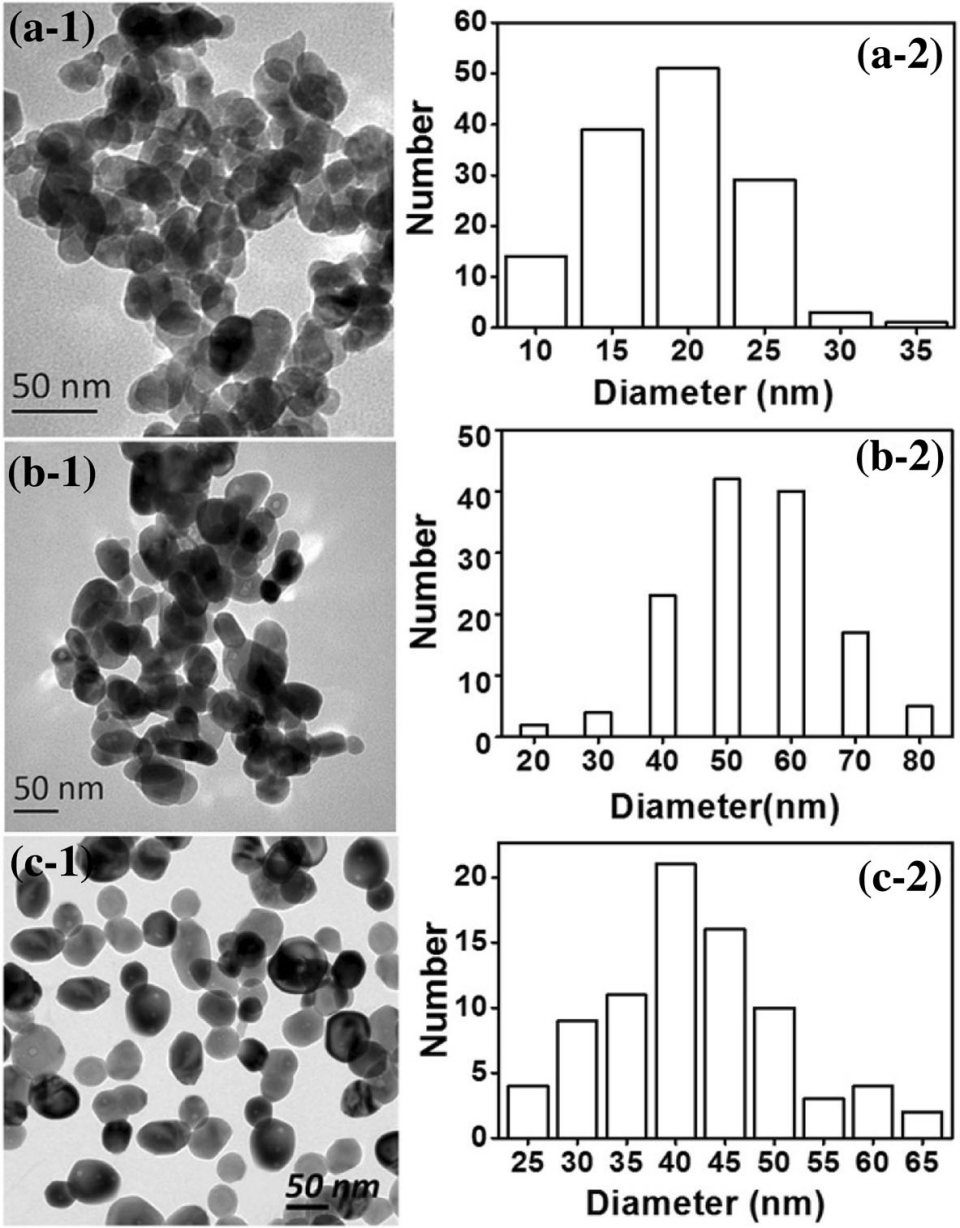
TEM images of ZnO nanoparticles obtained from 65% ZnCl_2_ aqueous solution obstruction by glycerol (**a**), starch (**b**), and cellulose (**c**) systems

In our previous study, ZnO nanoparticles were prepared via in situ synthesis of ZnO in dissolved starch or cellulose by a highly concentrated ZnCl_2_ aqueous solution [28, 29]. The glycerol system is compared with the dissolved starch or cellulose systems in this paper. As seen from Fig. 8, the ZnO nanoparticles were globular from all three processes. The ZnO nanoparticles obtained from dissolved starch or cellulose systems were 50–60 nm [28] or 40–50 nm [29], respectively, while those obtained from the glycerol system were 15–25 nm. More uniform and smaller ZnO nanoparticles could be prepared from the glycerol system. Although there are many hydroxyls on the molecular chains of starch and cellulose, there are three hydroxyls on the glycerol chain, viscosities of the zinc-cellulose or the zinc-starch composite are higher than that of the zinc-glycerol. So the colloid mill made the zinc-glycerol or ZnO-glycerol composite more easily into smaller droplets than ZnO-cellulose or ZnO-starch, resulting in smaller ZnO nanoparticles generated from the glycerol system.

In dissolved starch or cellulose systems, starch or cellulose were dissolved in a highly concentrated ZnCl_2_ aqueous solution, and then the ZnO-starch or cellulose nanocomposites were generated when the 40 wt% NaOH solution was added into ZnCl_2_ aqueous solution. The ZnO nanoparticles were obtained by calcining the dried ZnO-starch or cellulose nanocomposites. For the glycerol system, the glycerol was added into a concentrated ZnCl_2_ aqueous solution. The ZnO nanoparticles were easily obtained as the 50 wt% NaOH solution was added into glycerol-ZnCl_2_ aqueous solution. Therefore, the process using the glycerol system is easier and more cost-effective.

## Conclusions

Approximately 20 nm ZnO nanoparticles were prepared by a simple process in which hydroxide aqueous solutions were added to a glycerol-zinc chloride solution at room temperature to adjust the pH value to 12 with an extremely concentrated zinc source. The morphologies of ZnO could be controlled by the mole ratio of glycerol to Zn^2+^, the type of hydroxide, and the concentration of ZnCl_2_ aqueous solution. The glycerol acted as a stabilizer during the synthesis process; its blocking effect enhanced as the concentration of ZnCl_2_ aqueous solution or the mole ratio of glycerol to Zn^2+^ increased. The shape of ZnO changed from rods to globular, and the particle size decreased as the concentration of ZnCl_2_ aqueous solution or the mole ratio of glycerol to Zn^2+^ increased. Under optimal conditions, globular ZnO with an approximately 40–80 nm diameter was obtained from a ZnCl_2_ aqueous solution with a concentration of 65 wt% and a 1:1 mol ratio of glycerol to Zn^2+^. Moreover, the hydroxides also had an obvious influence on the size of ZnO particles. The granular ZnO nanoparticles with diameters approximately 20 or 50 nm could be generated from NaOH or KOH solutions, respectively, with saturation concentrations at room temperature. This study thus proposed a facile and size-controllable process for the synthesis of ZnO nanoparticles.

## Abbreviations

BD: 1,4-butanediol
DEG: Diethylene glycol
EG: Ethylene glycol
i-PrOH: Iso-propanol
PD: 1,3-propanediol
SEM: Scanning electron microscope
TEG: Tetraethylene glycol
TEM: Transmission electron microscope
XRD: X-ray diffraction patterns
ZnO: Zinc oxide
